# Vocal mother-offspring communication in guinea pigs: females adjust maternal responsiveness to litter size

**DOI:** 10.1186/1742-9994-5-13

**Published:** 2008-09-10

**Authors:** Melanie Kober, Fritz Trillmich, Marc Naguib

**Affiliations:** 1 Department of Animal Behaviour, University Bielefeld, P.O. Box 100 131, 33501 Bielefeld, Germany; 2 Netherlands Institute of Ecology (NIOO-KNAW), Department of Population Biology, P.O. Box 40, 6666 ZG Heteren, The Netherlands

## Abstract

**Background:**

In parent-offspring communication, vocal signals are often used to attract attention and offspring might call to induce parental behaviour. In guinea pigs (*Cavia aperea *f. *porcellus*) mothers wean larger litters later than small ones, but it is unknown whether this difference depends on processes induced during pregnancy or is influenced post-natally by the number of pups present. We here tested with playback-experiments using pup separation calls whether mothers with cross-fostered large experimental litters (four-pup-litters) were more responsive to offspring calls and maintained responsiveness for longer than mothers with small experimental litters (two-pup-litters). Mothers were tested when two pups were suckling i.e. when both teats were occupied.

**Results:**

Mothers of four-pup litters responded stronger to broadcast pup separation calls than those with two-pup litters. Additionally, we tested the mothers' responsiveness to pup separation calls in the absence of their pups on day 8 and 20 of lactation. Mothers of four-pup litters responded stronger and showed no decrease in responsiveness from day 8 to 20, whereas mothers of two-pup litters responded less and decreased responsiveness from day 8 to 20. Mothers of four-pup litters also weaned their pups 5 days later than those of two-pup litters.

**Conclusion:**

Measured by their response to pup calls and by time to weaning, guinea pig mothers adjust maternal responsiveness to litter size. This behaviour is likely to be an adaptive strategy in resource allocation during reproduction.

## Background

Offspring vary in their need and condition and parents must assess these parameters to provide optimal care. Therefore, offspring should communicate their needs to the parents and parents should act upon these signals. Studies on altricial animals show that offspring induce and maintain parental care by visual, acoustic, olfactory and tactile stimuli [1-6]. Maternal behaviour changes over time either due to a shift in maternal state or to changing stimulus characteristics of the young [3]. In rodents, maternal behaviour is influenced strongly by the age of pups and litter size [7,8]. Absence of sufficient stimuli may result in females abandoning offspring or even the complete brood [9,10]. In order to understand principles of resource allocation, maternal responsiveness to offspring stimuli and its change over time needs to be determined.

Rodents are an established model in such studies on maternal behaviour and parent-offspring conflict. Yet, most studies have been conducted on altricial species. Reproductive patterns differ for altricial and precocial rodent species particularly due to the fact that young of the latter begin early to contribute to their energy requirements by independent food intake [11]. In many species the sucking stimulus affects milk supply and the length of time to weaning [8,12]. For example in rats, lactation can be maintained far beyond normal weaning by repeatedly fostering younger pups to a mother [8]. However, in contrast to rats, the milk yield curve in guinea pigs (*Cavia aperea *f. *porcellus*) is rather fixed [13]. Mothers terminate lactation and only tend to prolong the lactation period slightly when given much younger foster pups [14]. Thus, these findings raise the question as to whether guinea pig mothers adjust their responses to experimentally manipulated offspring demand [14-16].

Previous studies showed that guinea pig females reduce milk output when litter size is reduced. In contrast, they do not increase milk yield proportionally to increases in litter size, at least not in litters consisting of more than three pups [13]. With an average litter size of more than three pups, but only two teats, competition between litter mates about access to milk is likely to occur and models of scramble competition describe pup-interactions better than honest signalling models [15]. Pups show moderately aggressive behaviour in the form of tussling. However, a previous study showed that pups do not succeed in getting access to a teat through tussling. Instead, hungrier pups respond faster to the presence of the mother and thereby gain preferential access to a teat [15].

One strategy by pups to obtain attention by the mother is to activate her by calling [17]. Vocal communication is important for precocial species where offspring actively moves around. Guinea pig mothers recognize their pups not only by olfactory cues but also by vocal cues and pup calls can induce female vocal responses [18,19]. As mothers of larger litters cannot nurse their complete litter simultaneously and as larger litters are weaned later [20], females with larger litters can be expected to be generally more responsive to their offspring for a longer period than females with small litters.

To test the hypothesis that mothers adjust responsiveness to calling pups according to litter size, we provided females with small or large litters through cross-fostering and conducted playback experiments with pup separation calls to test maternal responsiveness. We tested whether lactating mothers of larger (four-pup) experimental litters abandoned their two suckling pups more often than mothers of smaller (two-pup) experimental litters when another pup's separation calls were broadcast. We also tested the mothers' responsiveness to pup separation calls at different times in the lactation period (day 8 and 20; weaning occurs between day 25 and 30 depending on circumstances) and predicted that mothers with large experimental litters should respond stronger at both stages during lactation than mothers with small and less needy experimental litters.

## Methods

We conducted two playback experiments on outbred domestic guinea pigs at the University of Bielefeld, Germany. All subjects were kept indoors on a 14:10 (L:D) photoperiod at 20–23°C. Laboratory guinea pig chow (Höveler, Langenfeld, Germany) and water were provided *ad libitum*, supplemented with hay and fresh food. Females were allowed to breed in groups of two females and one male. 60 multiparous females were paired. Three days prior to the expected parturition date, females were kept singly in holding compartments (0.89 m × 0.89 m × 0.50 m). We created two- and four-pup litters by cross-fostering pups. To treat every litter equally, we cross-fostered pups even if a female's original litter size corresponded to the later experimental litter size, so that each female raised at least one foster pup and most females raised only foster pups and no own pup. We cross-fostered pups from litters born at the same day or on two subsequent days. Due to low synchrony of birth dates we could use only 28 females as experimental animals (13 litters of two and 15 litters of four pups). Original litter sizes did not differ significantly in experimental groups of two- and four-pup litters (Mann-Whitney *U*-test; *U *= 73.5, N_1 _= 13, N_2 _= 15, p = 0.27).

### Playback Stimuli

**Figure 1:**
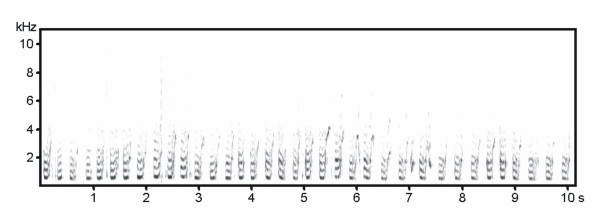
Sound spectrogram of a series of pup separation calls. Calls shown were recorded from an eight days old individual.

As playback stimuli we used pup separation calls [19]. For separation, a pup was removed from its holding compartment and placed in an enclosure (0.30 × 0.25 × 0.20 m) in an adjacent room. Separation lasted maximally 10 min. As this duration is within the range of natural feeding intervals, we did not provide food and water. We recorded pup separation calls (Fig. 1) using a Sennheiser ME 66/K6 directional microphone and a Sony TCD-D100 DAT-recorder. The microphone was located 30 cm above of the box. To avoid that potential developmental changes in call structure with age could confound responses [21], we recorded calls from pups of the same age as the pups of the experimental females at the time of the experiment (see below). Recordings were sampled at 44.1 kHz with a resolution of 16 bit and were transferred to a PC. We used Cool Edit 2000 (Syntrillium Corporation, Phoenix, USA) to generate playback files, and to normalize the recorded call series to the same peak amplitude to maintain natural variation in sound amplitude among calls. From the recordings we generated files with a 30 s call sequence (113 ± 6 calls within 30 s) that was repeated 15 times interspersed with 30 s silence so that each playback file had a duration of 15 min. This pattern of calling was within the range of natural calling sequences. In order to avoid pseudoreplication, calls from 72 different pups were used to produce 72 playback files so that each female received in each experimental part a file with separation calls from a different unfamiliar unrelated pup (experiment 1: n = 28; experiment 2: two-pup litter mothers: n = 11, four-pup litter mothers: n = 11, females were tested on day 8 (part 1) and on day 20 (part 2)). Unfamiliar pup separation calls were used because females with small litters did not have three pups which would have been required to generate a unique playback file for each of the three playbacks a female received. Since not all mothers with their pups participated in the experiments due to asynchrony in birth dates, we used preferentially these non-experimental pups for recordings. In a previous study we showed that females respond strongly to unfamiliar pup calls [19]. The use of calls recorded from pups of different litter sizes were balanced across experimental groups. Litter size of pups from which calls were recorded (small (one or two pups) versus larger (four or five pups)) did not affect female responses on neither day 8 nor on day 20 (Mann-Whitney *U*-tests; number of calls; all *U *> 7, all p > 0.5; approach, all *U *> 3, all p > 0.06).

### Experimental setup and playback experiments

All experiments were conducted indoors using a test-arena (1.48 × 0.98 × 0.47 m) in an experimental room acoustically separated from the colony room. In order to familiarize subjects to handling and the test environment, each subject was placed together with its pups in the test-arena for 15 to 30 min two times on the days prior to the experiments.

#### Experiment 1

**Figure 2:**
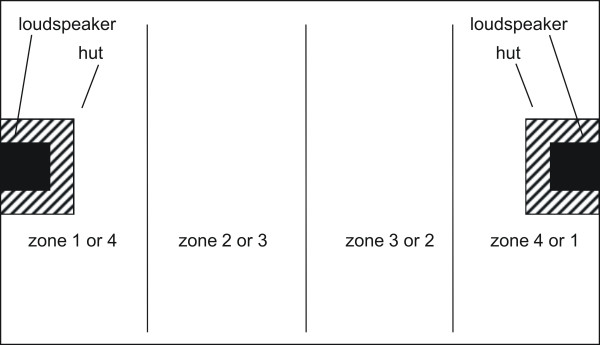
Experimental setup for experiment 1: The test-arena was equipped with huts for shelter on both sides of the test-arena and a loudspeaker placed on each hut. The zone closest to the loudspeaker that was used in a given trial was labelled as zone one and the most distant zone as zone four. Mother and pups could lie down for suckling anywhere in the arena. Stimuli were broadcast from the loudspeaker opposite to the animals' position.

Here we examined whether mothers of four-pup litters abandoned their suckling pups in response to playback more often than mothers of two-pup litters. We tested lactating females (n = 28) together with two of their pups on day 7 of lactation. On both sides, the test-arena was equipped with huts for shelter and a loudspeaker (Creative Inspire 2.1 2400) placed on each hut (Fig. 2). Since mother and pups could lie down for suckling anywhere in the box, we equipped both sides of the test-arena with a loudspeaker. Prior to the experiment all pups were separated from their mothers for 90 to 120 min to ensure that pups were hungry at the start of the experiment. Guinea pigs nurse every 20 to 60 min [20]. Thus the separation time is within natural feeding sequences since often not all pups have access to milk during one nursing bout. To maintain possibilities for some social interactions between the pups and their mother, all pups of the litter were jointly separated from their mother by a wire mesh in their holding compartment permitting visual, olfactory and acoustic contact with the mother, but without the possibility to suckle.

For the playback experiment, a mother was transferred together with two of its pups to the test-arena. In four-pup litters, we randomly selected two pups while the other two pups remained in their holding compartment. When both pups were suckling (mother in nursing position and both pups under the mother for at least 30 s) the playback session started. Playbacks were played directly from a PC and lasted for 15 min. The separation calls were broadcast from the loudspeaker opposite to the animals' position in the box.

#### Experiment 2

Here we tested whether mothers' responsiveness towards pup separation calls changes with offspring age in relation to experimental litter size of two or four pups. Mothers (without their pups) received a playback of pup calls on day 8 and day 20 of lactation; both parts followed the same general experimental procedure. The test-arena was equipped with one hut and one loudspeaker. Guinea pigs use huts for shelter, so we standardized the starting position of the female by providing a hut on the side of the test-arena opposite to the loudspeaker. We exchanged the position of hut and loudspeaker alternatingly between females, in order to control for possible side preferences. For playback, females were taken from their holding compartment and transferred directly to the test-arena. They were accustomed to the environment for 15 min after which the pre-playback period started. Each playback session consisted of a 15 min pre-playback period, and a 15 min playback period. Playbacks were played directly from a PC and females were under the hut at the onset of playback in all trials. In total, 22 females (two-pup litter mothers: N = 11, four-pup litter mothers: N = 11) received playback on day 8 and day 20. Six females had to be excluded because the playback trial either on day 8 or day 20 was disturbed, precluding a comparison between responses on day 8 and 20 in these cases.

In all experiments, calls were broadcast with a peak sound pressure level (SPL) of 75 dB as measured at 1 m with a sound level meter (Brüel & Kjær precision SPL meter 2233). This corresponds to the amplitude of natural calling as measured on 10 pups (unpublished data). After the experiments, subjects were returned immediately to their regular holding compartment.

### Weaning

To determine weaning age, pups were placed together with their mother in an elevated observation box (0.72 × 0.54 × 0.25 m) with a plexiglass bottom. Through the bottom it was possible for the observer to distinguish whether pups were suckling or only sitting under the mother. The subjects were familiarized to the box repeatedly during lactation. The observations took place once a day for 30 min from lactation day 18 on until a litter was weaned. When no suckling behaviour was recorded on three subsequent days, the first of these three days was defined as the day of weaning.

### Response measures and statistical analyses

Responses were recorded by direct observations. The observer [M.K.] sat silently in the same room sidewise to the centre of the test-arena and recorded the subject's behaviour (see below). For experiment 1 we counted how often mothers abandoned their two suckling pups. Since mothers and pups could reunite during the experiment, more than one abandoning of the pups per trial was possible. As responses in experiment 2 we recorded the subjects' vocal and spatial behaviour. In order to quantify the approach toward the loudspeaker, we divided the test-arena into four zones (Fig. 2). The observer recorded the subject's vocalizations and position at 10-second intervals over the 15 min playback period. Thus, we obtained 90 data points for vocalizations and for location within each playback session. For analyses we calculated (a) the number of intervals in which vocalizations occurred, (b) the latency to vocalize (in number of intervals), (c) the number of intervals females spent near the loudspeaker (in zone 4) and (d) the latency to approach zone 4 (in number of intervals). Responses were analyzed with Mann-Whitney-U tests and Wilcoxon matched pairs signed-ranks using SPSS 12.5. Two-tailed tests were used throughout. Weaning was analyzed using an analysis of variance, with original and experimental litter size as fixed factors.

## Results

**Figure 3:**
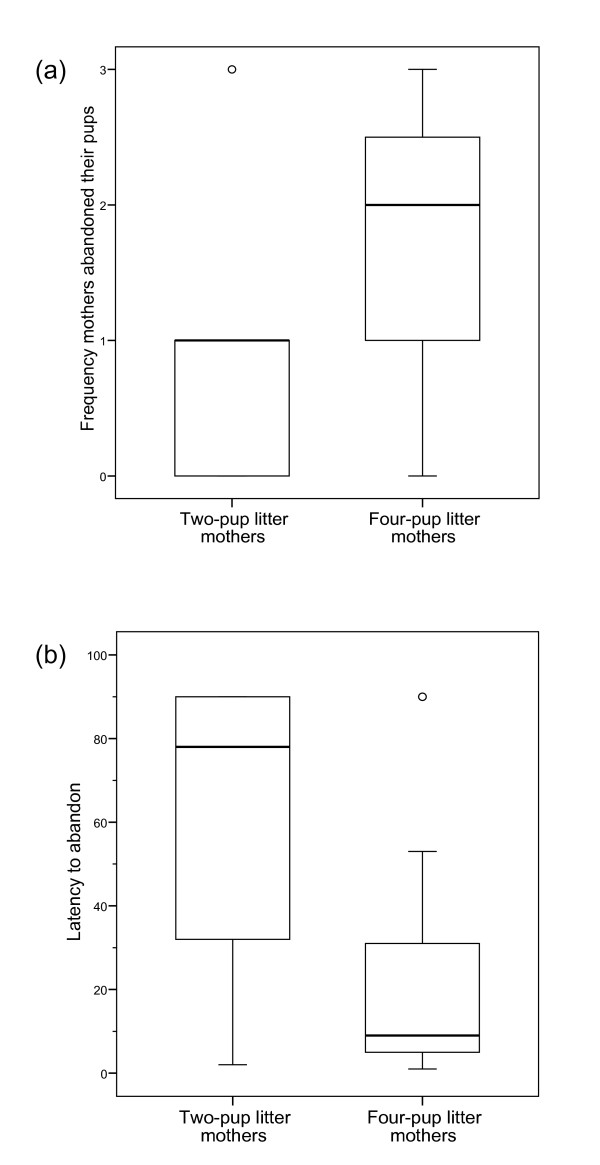
Responses of two-pup (*N *= 13) and four-pup (*N *= 15) mothers; (a) frequency to abandon the suckling pups, and (b) latency to abandon the suckling pups (10 sec intervals) during playback of another pup's calls (experiment 1). Each boxplot depicts median with inter-quartile range; whiskers extend to max. 1.5 times the inter-quartile range, outliers are shown as circles.

**Figure 4:**
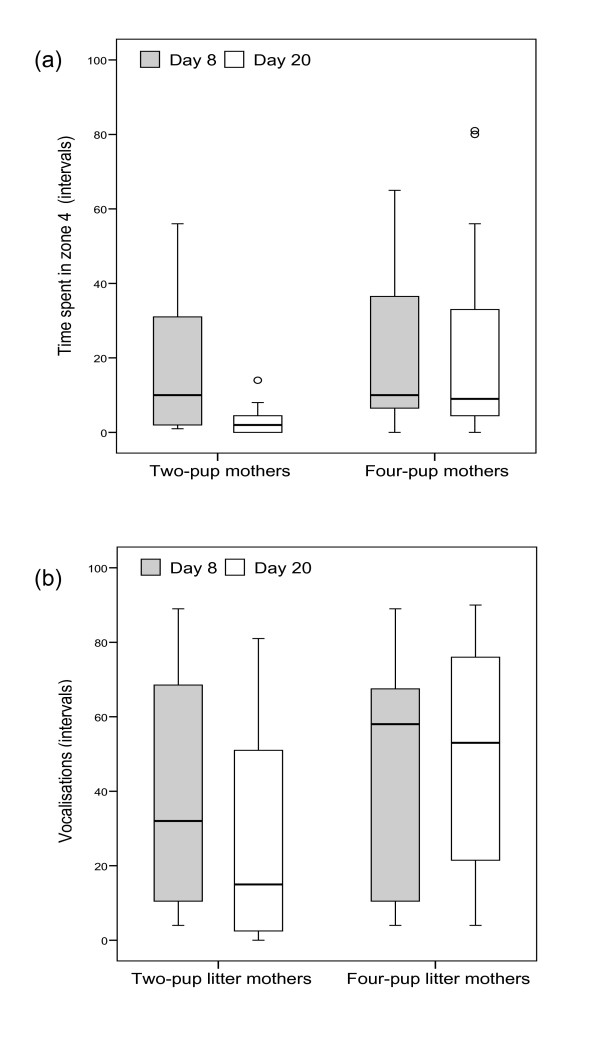
Responses of two-pup (*N *= 11) and four-pup litter (*N *= 11) mothers; (a) intervals spent in zone 4 (closest to the loudspeaker) and (b) intervals with vocalizations during playback of unfamiliar pup calls on day 8 and day 20 of lactation (experiment 2). Each box plot depicts median with inter-quartile range; whiskers extend to max. 1.5 times the inter-quartile range, outliers are shown as circles.

Mothers of four-pup litters abandoned their pups significantly more often and significantly earlier than mothers of two-pup litters (Figs. 3a, b; Mann-Whitney *U*-tests; abandoned: *U *= 47; N_1 _= 13, N_2 _= 15; p = 0.014; latency to abandon: *U *= 45.5; N_1 _= 13, N_2 _= 15; p = 0.015). Except for one female, four-pup-litter mothers abandoned their pups at least once per trial. Moreover, two-pup-litter mothers decreased the time spent near the loudspeaker significantly from day 8 to day 20 of the lactation period (Fig. 4a; Wilcoxon test; T = 3, N = 11, p = 0.01) whereas mothers of four-pup litters did not (Fig. 4a; Wilcoxon test; T = 34.5, N = 11, p = 0.91). Similarly, calling activity of two-pup-litter mothers decreased significantly from day 8 to day 20 (Fig. 4b; Wilcoxon test; T = 0, N = 11, p = 0.001), whereas that of four-pup litter mothers in contrast remained high (Fig. 4b; Wilcoxon test; T = 28.5, N = 11, p = 0.72).

In four-pup litters, neither mothers' latency to vocalize (Wilcoxon test; T = 10, N = 6, p = 0.13, five females had tied scores) nor latency to approach (Wilcoxon test; T = 17, N = 11, p = 0.95) differed significantly between day 8 and day 20. Mothers of two-pup litters had a higher latency to vocalize on day 20 than on day 8 (Wilcoxon test; T = 9, N = 11, p = 0.03) and tended to approach the speaker later on day 20 than on day 8 (Wilcoxon test; T = 5, N = 11, p = 0.078).

Large experimental litters were weaned on day 30 ± 4.2 (mean ± SD, N = 15), significantly later than small experimental litters, which were weaned on day 25 ± 3.1 (mean ± SD, N = 13). Mothers' original litter size did not influence weaning age significantly (ANOVA: experimental litter size, F_1,27 _= 10.26, p = 0.004; original litter size, F_1,27 _= 2.09, p= 0.12). When cross fostering, we controlled for differences in pup birth weigh among litters, so that pups of small and large experimental litters did not differ in mean birth weight (small litters: 83 ± 16 (mean ± SD), N = 13; large litters: 87 ± 20, N = 15; T-test: T = -0.503, p = 0.62). Consistent with earlier studies [15,22] we found lower growth rates in larger litters, which were also weaned later than smaller litters. Because of later weaning, pups of large litters did not differ from pups of small litters in body mass at time of weaning.

## Discussion

Mothers of large experimental litters abandoned their suckling pups more often than mothers of small experimental litters when separation calls of another pup were broadcast. Moreover, mothers of small litters decreased their responsiveness to pup calls from day 8 to day 20, whereas those of large litters remained strongly responsive. Mothers of small experimental litters also weaned the pups earlier than did mothers of large experimental litters. Thus, guinea pig mothers adjusted their level and time period of responsiveness to experimental litter size.

Earlier experiments had shown that guinea pig females responded, apart from a general decrease of nursing activity over time [23], little if at all, to changes in pup demand by increasing milk yield [13] or by adjusting nursing performance [14,16]. These experiments suggested that females pay surprisingly little attention to pup demand or pup state. However, these studies described nursing behaviour and milk yield and did not observe maternal behavioural responsiveness depending on litter size as our playback experiments did. Thus the playback experiments complement previous studies by showing that females indeed adjust behavioural responsiveness to litter size, even if the litter size they are rearing is not the one they had produced. These results also fit with earlier findings that larger litters are weaned later [15,16] and suggest that maternal motivation is increased through stimuli provided by larger litters. Previous studies on other rodents also had shown that maternal responsiveness depends on litter size. Maternal nest attendance decreased with increasing litter size in golden hamsters (*Mesocricetus auratus*) [24] and rats *(Rattus norvegicus*) [25] which may be due to the increased temperature in the nest or disturbances caused by the activity of many pups. Mongolian gerbil mothers (*Meriones unguiculatus*) with larger litters spent less time in the nest, but licked and sniffed more than mothers with small litters [26]. In contrast to these measures of maternal care, our playback experiments provide evidence for changes in maternal responsiveness to pup separation calls in relation to litter size.

The pup separation calls we tested, differ functionally from begging calls tested in other species. They are emitted only when pups are out of contact with their mother and are not given before and during suckling interactions. Playback experiments on pigs (*Sus scrofa domestica*) demonstrated a stronger response of mothers to needy piglets [27]. In these experiments, the smallest and slowest growing young in the litter which had just missed a nursing and were isolated in a relatively cool enclosure, called most intensely. Similarly, in birds a positive relationship between begging intensity and parental feeding rate could be found [4,28-31]. Great tit (*Parus major*) nestlings showed an increase in mean begging rates from experimentally reduced to enlarged broods. Their parents adjusted the feeding rates that were similar per nestling over three brood size [32].

Also, it has been suggested that piglet calls function mainly as a signal to the sow by piglets that are excluded from the current nursing episode [27]. Even though our experiments could be interpreted similarly, this function does not seem to apply to the separation calls as used in guinea pigs. Fey and Trillmich [15] never observed pups to utter separation calls when they were near their mother but had temporarily no access to the teats in litters of four. These pups would rather dig in under the mother, presumably to wait for an opportunity to access a teat. For female guinea pigs, finding lost pups may play a major role in protection of those pups and may also be important in thermoregulation, particularly for young pups that have only limited energy reserves to maintain thermoregulation. As energy input via milk plays only a minor role late in lactation [16], the benefits of lengthening the lactation period may rather be of social function or to support pups' thermoregulation.

Causally, differences in maternal responsiveness might have been linked to differences in hormonal state, as suggested by correlative studies in humans. In humans, maternal approach behaviour was directly associated with levels of cortisol and multiple regressions revealed that the infants' vocal behavior significantly predicted maternal level of cortisol [33]. Mothers showing the highest levels of maternal approach responses were those with a high cortisol concentration and either a positive maternal attitude, or a vocally more active infant. Based on these data, they speculate that for the new mother to exhibit a high level of responsiveness to her infant, she must attain a certain level of arousal [34], which can be produced by elevated cortisol [35]. Moreover, Fey & Trillmich [15] showed that maternal cortisol levels in guinea pigs decreased as pups grew older, and mothers rearing a litter of four pups maintained, although not significantly so, higher cortisol levels than those with litters of two pups. Thus, auditory stimuli may affect maternal responsiveness via the general adaptive functions of arousal and evocation of maternal behavior.

As predicted, guinea pig mothers with large litters actively interrupted nursing and responded to pup separation calls by approaching the loudspeaker whereas mothers of small litters most often did not. This indicates that mothers pay attention to litter size and do not respond when calling pups cannot be their own. We previously showed that females can recognize the calls of their own offspring [19] but nevertheless are responsive to calls of unfamiliar pups. This suggests that the costs of such false alarms are lower than the costs of missed detections [36]. Such a pattern of response is not unusual as also in ungulate hider species a similar unspecific response of mothers to separation calls has been reported [37]. In these species, females do not know the exact hiding location of their offspring and use vocalizations to reunite with the fawns [38,39]. Mule deer (*Odocoileus hemionus*) and white-tailed deer (*Odocoileus virginianus*) mothers defend their young vigorously against predators. The fawns' calls activated the mothers and in mule deer, females even responded to separation calls of white tailed deer calves [17,37]. These authors argue that it may pay more to mistakenly defend a foreign young than to lose the own fawn. However, as females also approached the loudspeaker in an aggressive manner when they were together with their own fawn, factors other than the separation from their fawn must also affect responsiveness to playback by female deer. In contrast to larger mammals, in guinea pigs and their wild ancestors, females have little possibility to actively defend their offspring, as mammalian and bird predators are able to kill the mother as well as the offspring. Therefore, the response by guinea pig mothers is likely to function to reunite with young gone astray rather than in directly defending them. Mothers with larger offspring numbers then presumably respond stronger than those with fewer offspring as it is more likely to lose young in larger litters. Reuniting with these young quickly and thereby preventing them from calling is likely to reduce the danger of attracting the attention of predators.

## Conclusion

Our findings demonstrate that guinea pig mothers adjust maternal responsiveness to increased litter size through an increased response to pup calls and an increase in time to weaning. This contradicts earlier findings which seemed to indicate that maternal responsiveness was determined largely during pregnancy and indicates a more complex mother-pup relation than documented previously.

## Authors' contributions

MK, FT and MN designed the experiments and wrote the manuscript. MK conducted the experiments and analyzed the data.
